# Elevated IL-6 Expression in Autologous Adipose-Derived Stem Cells Regulates RANKL Mediated Inflammation in Osteoarthritis

**DOI:** 10.3390/cells13242046

**Published:** 2024-12-11

**Authors:** Hyun-Joo Lee, Dae-Yong Kim, Hyeon jeong Noh, Song Yi Lee, Ji Ae Yoo, Samuel Jaeyoon Won, Yoon Sang Jeon, Ji Hoon Baek, Dong Jin Ryu

**Affiliations:** 1 Stem Cell R&D Center, N-BIOTEK, Inc., 104-706, Technopark Ssangyong 3Cha, 397, Seokcheon-ro, Bucheon-si 14449, Republic of Korea; nblhj@n-biotek.com (H.-J.L.); ceo@n-biotek.com (D.-Y.K.); cso@n-biotek.com (H.j.N.);; 2 N-BIOTEK, Inc., 402-803, Technopark, 655, Pyeongcheon-ro, Bucheon-si 14502, Republic of Korea; 3 Orthopedic Surgery, Inha University Hospital, Incheon 22332, Republic of Korea; 4 School of Medicine, Inha University, Incheon 22013, Republic of Korea

**Keywords:** autologous adipose-derived stem cell (ASCs), osteoarthritis (OA), interleukin-6 (IL-6), receptor activator of nuclear factor kappa-Β ligand (RANKL)

## Abstract

Interleukin-6 (IL-6) expression in mesenchymal stem cells (MSCs) has been shown to play a pivotal role in modulating cartilage regeneration and immune responses, particularly in the context of diseases that involve both degenerative processes and inflammation, such as osteoarthritis (OA). However, the precise mechanism through which IL-6 and other immune-regulatory factors influence the therapeutic efficacy of autologous adipose-derived stem cells (ASCs) transplantation in OA treatment remains to be fully elucidated. This study aims to investigate the relationship between IL-6 expression in autologous ASCs isolated from OA patients and their impact on immune modulation, particularly focusing on the regulation of Receptor Activator of Nuclear factor Kappa-Β Ligand (RANKL), a key mediator of immune-driven cartilage degradation in OA. Autologous ASCs were isolated from the stromal vascular fraction (SVF) of adipose tissue obtained from 22 OA patients. The isolated ASCs were cultured and characterized using reverse transcription polymerase chain reaction (RT-PCR), enzyme-linked immunosorbent assay (ELISA), and flow cytometry to the phenotype and immune regulatory factors of MSCs. Based on IL-6 expression levels, ASCs were divided into high and low IL-6 expression groups. These groups were then co-cultured with activated peripheral blood mononuclear cells (PBMCs) to evaluate their immune-modulatory capacity, including the induction of regulatory T cells, inhibition of immune cell proliferation, and regulation of key cytokines, such as interferon-gamma (IFN-γ). Additionally, RANKL expression, a critical factor in osteoclastogenesis and cartilage degradation, was assessed in both ASC groups. High IL-6-expressing ASCs demonstrated a significantly greater capacity to inhibit immune cell proliferation and IFN-γ production compared to their low IL-6-expressing counterparts under co-culture conditions. Moreover, the group of ASCs with high IL-6 expression showed a marked reduction in RANKL expression, suggesting enhanced potential to control osteoclast activity and subsequent cartilage defect in OA. Conclusion: Autologous ASCs with elevated IL-6 expression exhibit enhanced immunomodulatory properties, particularly in regulating over-activated immune response and reducing osteoclastogenesis through RANKL suppression. These findings indicate that selecting ASCs based on IL-6 expression could enhance the therapeutic efficacy of ASC-based treatments for OA by mitigating immune-driven joint inflammation and cartilage degradation, potentially slowing disease progression.

## 1. Introduction

Osteoarthritis (OA) is traditionally regarded as a degenerative joint disease primarily characterized by the progressive breakdown of cartilage, subchondral bone remodeling, and osteophyte formation. However, recent research underscores the significant role of inflammation in OA pathogenesis, redefining OA as a disease with both degenerative and inflammatory components [1,2]. Although OA is not classified as an inflammatory disease like rheumatoid arthritis, chronic inflammation contributes to its progression, symptomatology, and impact on joint function.

The inflammatory aspects of OA are evident in the presence of synovitis, where infiltrating immune cells, including macrophages, neutrophils, and lymphocytes, drive the release of pro-inflammatory cytokines such as interleukin-1β (IL-1β), tumor necrosis factor-alpha (TNF-α), and interleukin-6 (IL-6) [3]. These mediators exacerbate cartilage degradation by upregulating matrix metalloproteinases (MMPs) and aggrecanases, which break down the extracellular matrix (ECM) [4].

Conventional therapies of OA primarily focus on symptomatic relief, but there is an increasing demand for disease-modifying therapies that address the underlying causes of OA, such as inflammation and cartilage degradation [5,6]. Mesenchymal stem cells (MSCs) have emerged as promising candidates for regenerative medicine, particularly in OA, due to their ability to modulate immune responses, reduce inflammation, and promote tissue repair [7,8,9,10,11].

Among the various sources of MSCs, adipose-derived stem cells (ASCs) have gained significant attention because of their ease of isolation, abundance, and multi-potency [12]. ASCs are capable of differentiating into chondrocytes, osteoblasts, and adipocytes, making them potential candidates for cartilage repair [13,14]. Additionally, ASCs exhibit potent immunomodulatory effects by secreting anti-inflammatory cytokines and promoting the expansion of regulatory T cells (Tregs), which are essential for maintaining immune homeostasis and attenuating inflammatory responses [15,16]. However, variations in the therapeutic efficacy of ASCs, influenced by factors such as donor age, health status, tissue origin, and specific cytokine expression profiles, remain under active investigation. 

One cytokine of particular interest in OA is interleukin-6 (IL-6), a pleiotropic pro-inflammatory cytokine that is elevated in OA joints and associated with disease progression [17]. IL-6 plays a dual role in inflammation, contributing to both pro-inflammatory and anti-inflammatory responses depending on the context [18]. Elevated IL-6 levels promote osteoclastogenesis by upregulating the receptor activator of nuclear factor-kappa B ligand (RANKL), a key mediator of bone resorption and joint degradation in OA [19,20]. However, IL-6 has been implicated in cartilage repair processes. It has been shown to promote chondrogenic differentiation of MSCs through the activation of the IL-6/STAT3 signaling pathway, suggesting a potential role in maintaining cartilage homeostasis and supporting self-repair mechanisms in OA [21]. Given these factors, understanding how IL-6 expression in ASCs influences their regenerative and immunomodulatory capacities is critical for optimizing their therapeutic use in OA.

This study aims to elucidate the influence of IL-6 expression on the functional properties of ASCs derived from osteoarthritic adipose tissue, with the goal of optimizing their therapeutic potential for OA. Specifically, we explored how IL-6 expression affects ASC-mediated immune modulation, including Treg induction and RANKL inhibition. By dividing ASCs into two groups based on IL-6 expression levels (high and low), we analyzed their ability to suppress immune cell proliferation and inflammatory cytokine production in a co-culture system with PBMCs. Additionally, we assessed the impact of IL-6 expression on RANKL levels, a critical driver of osteoclastogenesis and joint destruction in OA, using FACS analysis to quantify RANKL expression in immune and stem cell populations. These findings provide new insights into the role of IL-6 in regulating ASC function and offer potential strategies for enhancing the efficacy of ASC-based therapies for OA.

## 2. Materials and Methods

### 2.1. OA Patients and Adipose Tissues Obtained

This procedure was conducted as a preparatory step for the intra-articular injection of autologous ASCs in patients with knee OA. After obtaining informed consent, the adipose tissue was harvested from the abdomen of 22 patients with knee OA (K-L grade II: 9 patients, K–L grade III: 8, K-L grade IV: 5 patients). After administering local anesthesia to the periumbilical skin tissue using 5 cc of 1% lidocaine (Huons, Sungnam-si, Kyunggi-do), a tumescent solution (0.9% normal saline 250 mL (JW medicine, Gwacheon-si, Kyunggi-do, Republic of Korea) + 1% lidocaine 20 mL (Huons, Sungnam-si, Kyunggi-do, Republic of Korea) + 1:1000 epinephrine 1 mL (DAI HAN PHARM CO, Seoul, Republic of Korea) + 8.4% sodium bicarbonate 5 mL (Huons, Sungnam-si, Kyunggi-do) was injected into the subcutaneous adipose tissue for even distribution. After 10 min for tissue emulsification, a liposuction cannula was inserted into the subcutaneous fat layer with 0.7 cm mini skin-incision, and suction was applied at 150 mmHg pressure. Care was taken to handle the tissue gently to minimize bleeding, and a total of 70–100 cc, including blood and tumescent solution, was collected. This procedure was conducted by one orthopedic surgeon (DJR). This protocol was approved by the investigational review board of our institution (Inha Univ Hospital IRB 2023-06-020).

### 2.2. Isolation and Characterization of Adipose-Derived Mesenchymal Stem Cells (ASCs)

The ASCs were isolated from adipose tissue obtained at INHA University Hospital. The adipose tissue was rinsed three times with phosphate-buffered saline (PBS) (Lonza, Walkersville, MD, USA) and enzymatically digested with 0.1% (*w*/*v*) Type I collagenase (Nordmark Pharma GmbH, Uetersen, Germany) at 37 °C for 45 min. Following digestion, the remaining undigested tissue and oil were removed by filtration through a 100 μm size mesh, followed by centrifugation at 1500 rpm for 5 min. The cell pellet was resuspended in primary culture medium consisting of α-Minimun Essential Medium (α-MEM) (Welgene, Gyeongsan, Gyeongsangbuk-do, Republic of Korea), 1% fetal bovine serum (FBS) (Gibco, Grand Island, NY, USA). The cells were seeded at a density of 1 × 10^4^ cells/cm^2^ in fresh medium and maintained in a humidified incubator at 37 °C with 5% CO_2_. After 24 h, non-adherent cells were removed, and the medium was replaced with a complete culture medium consisting of α-MEM (Welgene, Gyeongsan, Gyeongsangbuk-do, Republic of Korea), 9% FBS, and 1 ng/mL bFGF (R&D Systems, Minneapolis, MN, USA), with medium changes performed every 2–3 days. The differentiation potential of ASCs into chondrocytes, adipocytes, and osteocytes was assessed using the Mesenchymal Stem Cell Identification Kits (R&D Systems, Minneapolis, MN, USA) according to the manufacturer’s instructions over 21 days. 

### 2.3. Immunofluorescence (IF) Analysis 

The differentiation potential of ASCs into chondrocytes, adipocytes, and osteocytes was assessed using the Mesenchymal Stem Cell Identification Kits (R&D Systems, Minneapolis, MN, USA) according to the manufacturer’s instructions over 21 days. Adipogenic differentiation was confirmed by immunostaining with an anti-FABP4 antibody, osteogenic differentiation with an anti-hOsteocalcin antibody, and chondroblast differentiation with an anti-hAggrecan antibody (1:100; all from R&D Systems, MN, USA) for 24 h, followed by 2 h of incubation with NL557-conjugated donkey anti-goat secondary antibodies (1:1000; Molecular Probes, Eugene, OR, USA). In addition, to analyze the expression of IL-6Rα of ASCs, the cells were stained with anti-IL-6Rα antibody (1:50; Santa Cruz Biotechnology, Santa Cruz, CA, USA) for 24 h, followed by 4 h of incubation with NL557-conjugated donkey anti-mouse secondary antibodies (1:1000; Molecular Probes, Eugene, OR, USA). For counterstaining, the nuclei were stained with DAPI. The staining results were photographed by TS100 Nikon inverted microscope (Tokyo, Japan). 

### 2.4. Quantitative Real-Time PCR

Total RNA was extracted following the manufacturer’s instructions in the RNeasy Plus Mini kit (QIAGEN Inc., Germantown, MD, USA). cDNA synthesis was carried out using a high-capacity cDNA reverse transcription kit (Applied Biosystems, Waltham, MA, USA) following the manufacturer’s protocol. Quantitative real-time PCR (qRT-PCR) reactions were conducted on a QuantStudio 3 Real-Time PCR (Applied Biosystems, MA, USA), using SYBR Green Universal Master Mix (Applied Biosystems, Waltham, MA, USA) and gene-specific primers. The primer sets utilized for gene amplification were as follows: IL-6 (P211161, Bioneer, Daejeon, Republic of Korea), TGF-β1 (P256170, Bioneer, Daejeon, Republic of Korea). Gene expression fold changes were determined using the comparative Ct method (2-ΔΔCt), which compares the target gene normalized to GAPDH.

### 2.5. Conditioned Medium Collection

ASCs were seeded at a density of 5 × 10^5^/well in a 6-well culture plate and incubated for 1 day at 37 °C with 5% CO_2_ in a humidified chamber. Then, ASCs were washed 3 times using PBS and replaced with Phenol red-free DMEM (Welgene, Gyeongsan, Gyeongsangbuk-do, Republic of Korea). The cells were cultured in hypoxic conditions (2% O_2_, 5% CO_2_) for 2 days. The conditioned media samples were collected, centrifuged at 1500 rpm for 5 min to remove debris, and then frozen in aliquots at −70 °C.

### 2.6. Enzyme-Linked Immunosorbent Assay (ELISA)

Concentrations of IL-6, CCL2, and TGF-β1 in supernatants of ASCs were measured by enzyme-linked immunosorbent assay (ELISA) kit (RayBiotech, Peachtree Corners, GA, USA) according to the manufacturer’s instructions.

### 2.7. Isolation and Stimulation of PBMC

To assess the in vitro immunosuppressive activity of ASCs, PBMCs were isolated from buffy coats of healthy male donors using Ficoll–Paque (GE Healthcare, Milwaukee, WI, USA). For the Treg induction analysis, PBMCs (2.5 × 10^5^ cells/well) from two donors were co-cultured with ASCs (5 × 10^4^ cells/well) in a 24-well plate in a mixed lymphocyte reaction (MLR) setting for three days in RPMI 1640 medium supplemented with 10% FBS, 2 mM L-glutamine, and 100 U/mL penicillin. For cytokine secretion and activation marker analysis, PBMCs (5 × 10^5^ cells/well) were co-cultured with adherent ASCs (5 × 10^4^ cells/well) in 24-well plates and treated with 5 µg/mL of PHA (Sigma-Aldrich, St. Louis, MO, USA) for 72 h. Cells and supernatants were then collected for analysis. Additionally, for the proliferation assay, PBMCs were labeled with CFSE (Invitrogen, St. Louis, MO, USA) at a final concentration of 5 µM.

### 2.8. Flow Cytometry

Cell surface staining was performed using allophycocyanin (APC)-cy7 conjugated CD4 (BioLegend, San Diego, CA, USA), fluorescein isothiocyanate (FITC)-conjugated CD25 (Biolegend, San Diego, CA, USA), or phycoerythrin (PE)-conjugated RANKL (Biolegend, San Diego, CA, USA) for 20 min at 4 °C in the dark to analyze the expression of T cells. After the stained cells were washed with PBS, intracellular staining was performed to analyze the Treg population. According to the manufacturer’s instructions, the cells were fixed and permeabilized using a Transcription Factor Staining Buffer Set kit (eBioscience, San Diego, CA, USA) and stained with PE-conjugated foxp3. 

### 2.9. Cytometric Bead Array 

The concentrations of interferon-γ (IFN-γ), tumor necrosis factor-α (TNF-α), Interleukin-10 (IL-10), and IL-17A in the culture supernatants of ASCs and PBMCs in co-culture were measured using a custom-made LEGENDPlex kit (BioLegend, San Diego, CA, USA), according to the manufacturer’s instructions. Measurements were performed using a flow cytometer (Beckman Coulter, Brea, CA, USA). Cytokine concentrations are expressed as pg/mL.

### 2.10. Statistical Analysis

All data are presented as the mean ± standard error of the mean. Statistical differences were assessed by Student’s *t*-test or one-way ANOVA using GraphPad Prism (version 8; Graph Pad Software, San Diego, CA, USA). The statistical values are detailed in the figure legend. 

## 3. Results

### 3.1. Characterization of ASCs Isolated from Osteoarthritis Patients Reveals Mesenchymal Stem Cell-like Properties

**Figure 1:**
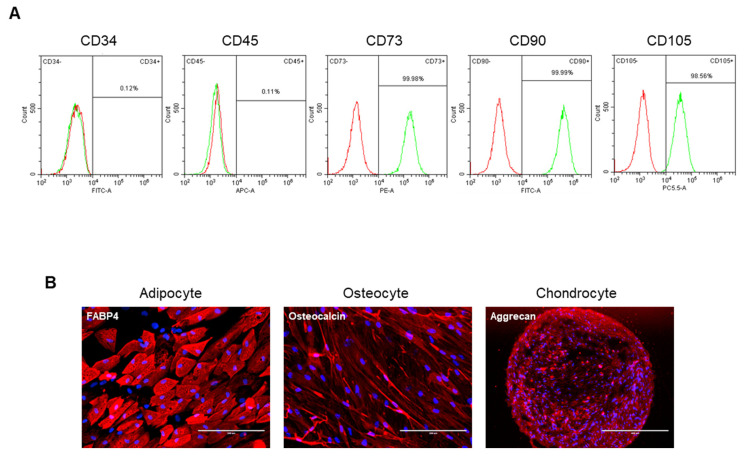
The characterization of ASC-derived OA patients. (**A**) FACS analysis showing the expression of MSC characterization markers in representative ASCs used in this study. Green histograms represent staining with isotype-matched control antibodies, while red histograms depict the specific expression of each indicated marker. (**B**) Immunofluorescence staining for FABP4, Osteocalcin, and Aggrecan in ASCs induced to undergo adipogenic, osteogenic, and chondrogenic differentiation, respectively. DAPI was used for nuclear staining. Scale bars: FABP4, 400 μm; Osteocalcin, 200 μm; Aggrecan, 400 μm).

In this study, we isolated autologous adipose-derived stem cells (ASCs) from 22 OA patients. Detailed patient demographics and clinical information are presented in Table 1. To further characterize the ASCs, we analyzed the surface antigen markers of a representative ASC sample using flow cytometry (Figure 1). The cells were positively labeled for common mesenchymal stem cell (MSC) markers, including CD73, CD90, and CD105, while showing negative expression for hematopoietic markers (CD45 and CD34). Additionally, the differentiation potential of these ASCs was assessed, confirming their ability to differentiate into osteogenic, adipogenic, and chondrogenic lineages under specific culture conditions. These results confirm the mesenchymal origin and multipotency of the isolated ASCs from OA patients.

### 3.2. IL-6 Expression in ASCs from Osteoarthritis Patients and Immune Modulation 

**Figure 2:**
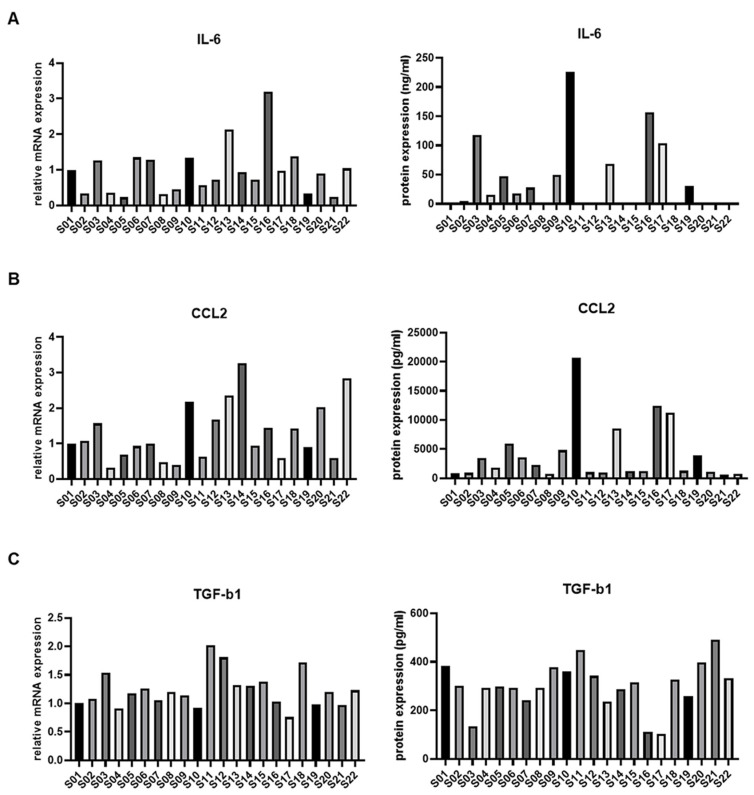
Gene and protein expression analysis of ASCs related to immunoregulatory factor, IL-6, TGF-b, and CCL2. The analysis was conducted on ASCs isolated from 22 OA patients. Gene and protein expression levels are presented relative to the levels observed in ASCs isolated from patients S01 to S22. Expression of (**A**) IL-6, (**B**) TGF-β, and (**C**) CCL2 in ASCs. The left panel shows mRNA expression levels, and the right panel presents protein expression both relative to gene and protein levels in ASCs isolated from patients S01 to S22.

To examine the expression patterns of immune regulatory factors in ASCs from OA patients, we analyzed IL-6, CCL2, and TGF-β, all of which play critical roles in immune regulation and inflammation in OA pathogenesis. To assess the expression patterns of these mediators in ASCs from OA patients, we evaluated the expression of mRNA expression and protein synthesis levels of IL-6, CCL2, and TGF-β (Figure 2). Culturing ASCs under hypoxic conditions allowed us to obtain precise measurements of protein expression. Among the 22 ASC samples, IL-6 demonstrated the most pronounced variability, suggesting its potential impact on immune modulation and ASC functionality. To further investigate the role of IL-6 expression, we performed immunofluorescence (IF) analysis to examine the expression of IL-6 receptor alpha (IL-6Rα), which is known to be induced through autocrine signaling in cells exhibiting elevated IL-6 levels [18,22]. The results revealed that IL-6Rα expression was higher in the high IL-6 group compared to the low IL-6 group, confirming that IL-6 signaling is activated under hypoxic conditions (Supplementary Figure S1). Based on these results, we further explored the role of IL-6 in the characterization of ASCs.

### 3.3. Grouping of ASC Based on IL-6 Expression and Its Impact on MSC Characteristics

**Figure 3:**
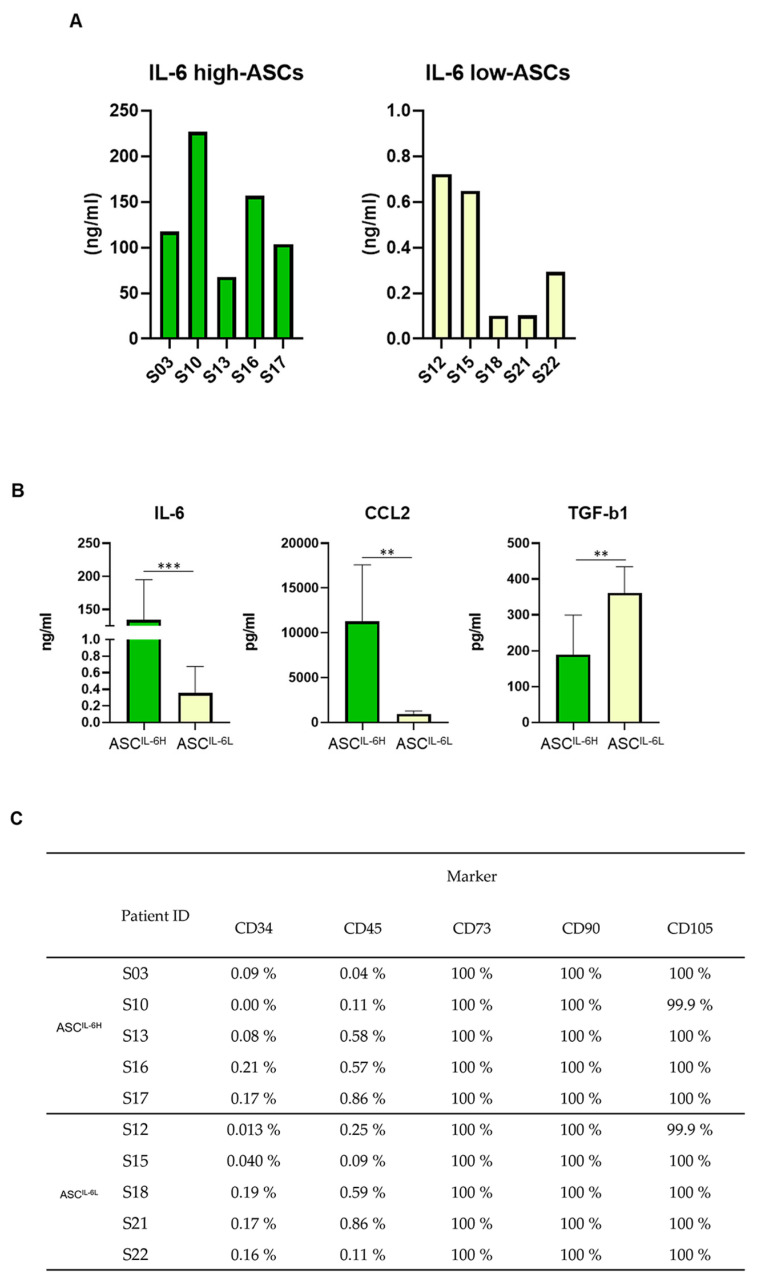
Comparative analysis of IL-6 expression in ASCs from two groups of osteoarthritis (OA) patients based on IL-6 levels. Based on the IL-6 protein expression, the ASCs of OA patients were divided into two groups: ASCs of 5 patients with high IL-6 expression (ASC^IL-6H^) and five patients with low IL-6 expression (ASC^IL-6L^). (**A**) Individual IL-6 protein expression values in each patient, divided into the ASC^IL-6H^ and ASC^IL-6L^ groups. (**B**) The average levels of protein expression for IL-6, TGF-β, and CCL2 in the ASC^IL-6H^ and ASC^IL-6L^ groups. (**C**) Table summarizing MSC characterization marker expression for the ASC^IL-6H^ and ASC^IL-6L^ groups. The experiments were conducted in triplicates. The results are shown as the mean ± SD. ** *p* < 0.01, *** *p* < 0.001.

To investigate the potential influence of IL-6 expression on the fundamental characteristics of ASCs in OA patients, 22 OA patients were screened for IL-6 expression. Based on these results, we classified the ASCs into two groups: the IL-6 high group; ASC^IL-6H^, consisting of ASCs from the top 5 patients with the highest IL-6 expression, and the IL-6 low group; ASC^IL-6L^, consisting of ASCs from the five patients with the lowest IL-6 expression (Figure 3A). The expression patterns of MSC surface antigen markers, along with immune-regulating factors, CCL2 and TGF-β, were analyzed in the two divided groups. CCL2 exhibited a similar trend as IL-6, with significantly higher expression in the ASC^IL-6H^ compared to the ASC^IL-6L^. Conversely, TGF-β expression showed an opposite pattern, with significantly lower levels in the ASC ^IL-6H^ (Figure 3B). Despite these differences in cytokine expression, there were no notable differences in the basic MSC surface marker profile between the two groups (Figure 3C). Given these findings, we proceeded to further experiments aimed at evaluating the immune-modulating capabilities of these ASCs, particularly their ability to regulate immune activity in OA.

### 3.4. Impact of IL-6 Expression on Treg Induction by ASCs

To clarify the immunomodulatory effect of ASCs, we investigated the ability of the ASC^IL-6H^ and ASC^IL-6L^ to induce regulatory T cells (Tregs) in co-culture in allogeneic mixed lymphocyte reaction (MLR). The Treg induction capacity of ASCs was evaluated using mixed lymphocyte reaction (MLR) conditions, known to induce Tregs to determine whether IL-6 expression levels in ASCs influence this property [23].

**Figure 4:**
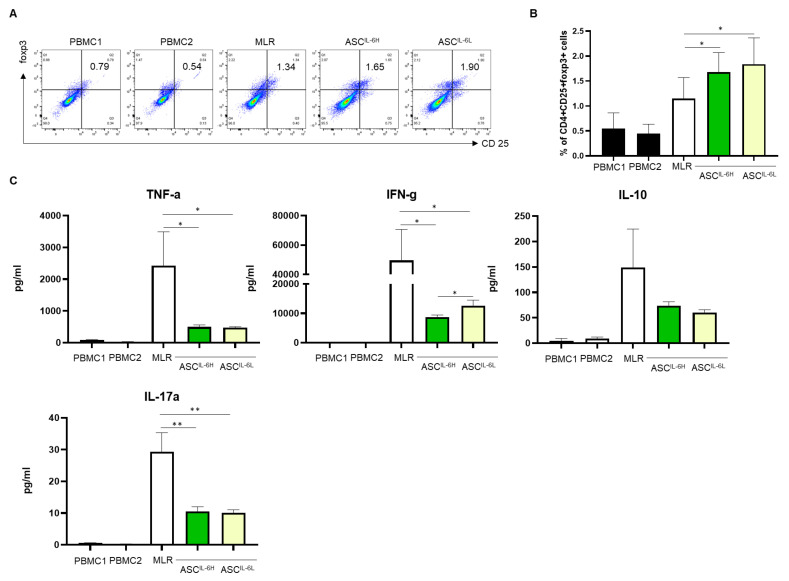
Comparison of Regulatory T Cell Induction between ASC^IL-6H^ and ASC^IL-6L^. (**A**) Flow cytometry analysis of CD4^+^CD25^+^FoxP3^+^ regulatory T cells (Tregs) under allogeneic mixed lymphocyte reaction (MLR) cultures with the addition of ASC^IL-6H^ and ASC^IL-6L^. Five ASCs from each group were cultured under MLR conditions to determine Treg inductivity. (**B**) In the presence of ASC^IL-6 H^ and ASC^IL-6L^, the bars represent the median and range of results obtained from the five ASCs in each group co-cultured under MLR conditions. (**C**) Cytokine levels in the supernatant of MLR cultures, as measured by CBA assay. The levels of IFN-γ, TNF-α, IL-10, and IL-17 were quantified. The experiments were conducted in triplicates. The results are shown as the mean ± SD. * *p* < 0.05, ** *p* < 0.01.

Co-culture of ASCs in the MLR condition resulted in a moderate but significant increase in the percentage of CD4^+^CD25^+^FoxP3^+^ Tregs in both the ASC^IL-6H^ and ASC^IL-6L^ groups compared to the Treg population under the MLR culture condition. However, there was no statistically significant difference in the Treg induction capacity between ASC^IL-6H^ and ASC^IL-6L^ (Figure 4A,B). Next, we further examined the secretion of key cytokines, including IFN-γ, TNF-α, IL-10, and IL-17, in the MLR co-culture supernatants. While both ASC^IL-6H^ and ASC^IL-6L^ led to a prominent reduction in the pro-inflammatory cytokines IFN-γ and TNF-α compared to the MLR control, the ASC^IL-6H^ significantly suppressed the production of IFN-γ (Figure 4C). On the other side, the ability of ASCs to induce IL-10, an anti-inflammatory cytokine typically linked to regulatory T-cell activity, was not significantly enhanced in either group. Overall, these results suggest that IL-6 expression in ASCs may modulate the immune regulatory network through mechanisms that are independent of regulatory T cells.

### 3.5. Impact of IL-6 Expression on the Suppression of PHA-Induced PBMC Proliferation by ASCs

**Figure 5:**
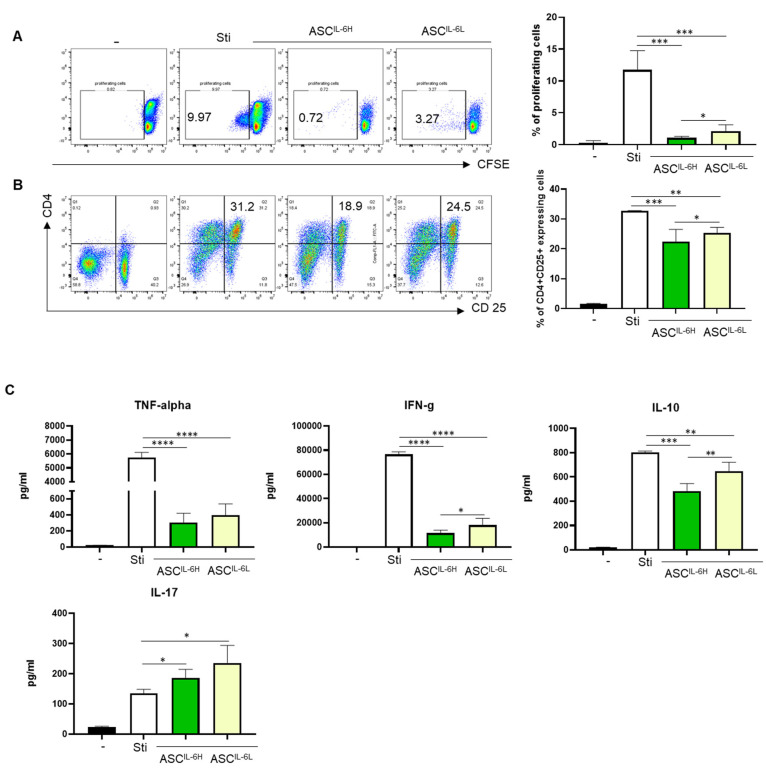
Comparison of Immunomodulatory effect between ASC^IL-6H^ and ASC^IL-6L^. PBMCs stimulated with phytohaemagglutinin (PHA) were cultured alone or in the presence of the five ASCs from each group, ASC^IL-6H^ and ASC^IL-6L^. (**A**) After 72 h of co-culture, the proliferating cells and CD25 expression were analyzed by flow cytometer. (**B**) In the presence of ASC^IL-6H^ and ASC^IL-6L^, the bars represent the median and range of results obtained from the five ASCs in each group co-cultured with PHA-stimulated PBMCs. (**C**) The production of IFN-g, TNF-a, IL-10, and IL-17 in the supernatant of PHA stimulation was analyzed by CBA assay. The experiments were conducted in triplicates. The results are shown as the mean ± SD. * *p* < 0.05, ** *p* < 0.01, *** *p* < 0.001, **** *p* < 0.0001.

To further explore the immunomodulatory properties of ASCs based on IL-6 expression levels, we evaluated the effect of ASC^IL-6H^ and ASC^IL-6L^ on the proliferation and activation of PHA-stimulated PBMCs. PBMCs from healthy donors were activated with 5 μg/mL of PHA, resulting in a significant upregulation of their proliferation compared to untreated controls. After three days of culture, PBMC proliferation dramatically increased. We then assessed the ability of both ASC^IL-6H^ and ASC^IL-6L^ to suppress this PHA-induced proliferation (Figure 5A). Our results demonstrated a statistically significant difference in the suppressive effects on PHA-induced PBMC proliferation between the two ASC groups.

ASC^IL-6H^ exhibited a significantly greater ability to suppress PHA-induced PBMC proliferation compared to ASC^IL-6L^. The suppressive effect of ASC^IL-6H^ was not only observed in the reduction of overall proliferation but also in the expression of T lymphocyte activation markers, CD25, and inflammatory cytokines compared to ASC^IL-6L^ (Figure 5B). 

Moreover, the production of inflammatory cytokines, including TNF-α and IFN-γ, was markedly suppressed in the presence of ASC^IL-6H^. Remarkably, IFN-γ was significantly inhibited (Figure 5C). As shown in Figure 4C, this result was consistent with findings observed under MLR conditions. These findings suggest that ASCs with higher IL-6 expression may have stronger immunosuppressive potential, which could be highly beneficial in treating inflammatory conditions.

### 3.6. Impact of IL-6 Expression on the Suppression 

**Figure 6:**
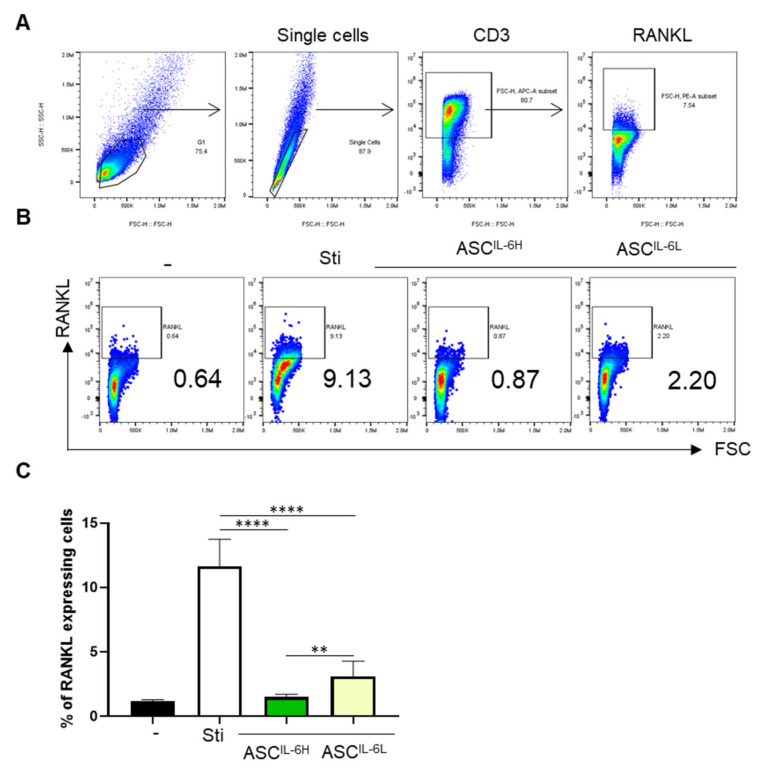
Regulatory Effect on RANKL of immune cells by IL-6 Expression in ASCs. PBMCs stimulated with cultured alone PHA or in the presence of the five ASCs from each group: ASC^IL-6H^ and ASC^IL-6L^. After 72 h of co-culture, The RANKL expression of T cells was analyzed by flow cytometer. (**A**) The gating strategy for RANKL expression of CD3^+^ T cells under PHA stimulation. (**B**) Flow cytometry analysis for RANKL expression of CD3^+^ T cells. (**C**) In the presence of ASC_IL-6H and ASC_IL-6L, the bars represent the median and range of results obtained from the five ASCs in each group co-cultured with PHA-stimulated PBMCs. The experiments were conducted in triplicates. The results are shown as the mean ± SD. ** *p* < 0.01, **** *p* < 0.0001.

Activated immune cells, especially T cells, express RANKL, a member of the TNF family, which promotes osteoclastogenesis and cartilage destruction. Therefore, the enhanced RANKL expression due to excessive immune activation can lead to increased bone resorption and joint damage in diseases such as OA and rheumatoid arthritis [24]. Given that activated immune cells express RANKL, which is linked to osteoarthritis aggravation, we investigated whether ASC-expressed IL-6 could modulate RANKL expression [23]. To investigate how ASC^IL-6H^ and ASC^IL-6L^ affect this response, we characterized the expression of RANKL in PHA-activated CD3^+^ T cells. PBMCs were gated on single cells of lymphocytes, and RANKL^+^ cells were identified from CD3^+^ cells (Figure 6A). Our results revealed the stimulation of PBMCs with PHA resulted in a significant upregulation of RANKL expression and the ability of ASC^IL-6H^ and ASC^IL-6L^ to suppress PHA-induced RANKL expression in PBMCs (Figure 6B). ASC^IL-6H^ exhibited a much stronger inhibitory effect on RANKL expression compared to ASC^IL-6L^ (Figure 6C). This highlights the enhanced ability of ASC^IL-6H^ to regulate RANKL expression, which could have important implications for controlling osteoarthritis progression.

## 4. Discussion

In this study, we investigated the effects of IL-6 expression in ASCs from OA patients on immune regulatory functions. By categorizing ASCs into two groups based on IL-6 expression (ASC^IL-6H^ and ASC^IL-6L^), we evaluated their immunomodulatory properties, including Treg induction, suppression of activated immune cell proliferation, T cell activation markers, and the expression of RANKL. Our findings provide valuable insights into how IL-6 may influence ASC function, highlighting its potential implications for osteoarthritis therapy. 

Mesenchymal stem cells are known for their ability to induce Tregs, a critical function in immune modulation [25]. In our study, we observed that both IL-6 high and low ASCs induced a slight increase in CD4^+^CD25^+^FoxP3^+^ Treg populations when co-cultured with PBMCs. This effect is consistent with previous reports showing that ASCs promote Treg generation, which contributes to their anti-inflammatory properties [26]. However, we observed no significant difference in Treg induction between the ASC^IL-6H^ and ASC^IL-6L^, suggesting that IL-6 may not be a critical factor in regulating Treg generation. This finding supports earlier work by Melief et al. [27], who also found that Treg induction was largely independent of IL-6 levels in MSCs. 

Our findings also align with previous reports that demonstrate impaired IL-10 production capabilities of ASCs derived from osteoarthritis (OA) patients, as demonstrated in studies like Skalska and Kontny et al. [28]. The reduction in anti-inflammatory cytokines, along with the observed increase in IL-17A secretion, is consistent with those patterns. However, while Skalska and Kontny et al. did not observe significant immunosuppressive effects in OA-ASCs, our study revealed marked suppression of immune activation in both ASC^IL-6H^ and ASC^IL-6L^. This suggests that ASCs may utilize alternative mechanisms beyond IL-10-producing Tregs to suppress pro-inflammatory cytokines like IFN-γ and TNF-α. The concurrent suppression of both pro-inflammatory and anti-inflammatory cytokines under immune activation supports the possibility that other factors, such as IL-10-independent Tregs, may mediate immune suppression through pathways like TGF-β-mediated signaling or direct cell contact. 

IL-6 is typically recognized as a pro-inflammatory cytokine that promotes the differentiation of Th17 cells, contributing significantly to autoimmune conditions like rheumatoid arthritis through its role in inducing IL-17A secretion [29]. However, the function of IL-6 expressed in MSCs differs from that in immune cells. Previous studies have demonstrated that IL-6 can contribute to immunosuppressive functions and cellular proliferation, crucial for therapeutic applications in inflammatory diseases [30,31,32]. In our study, IL-6-highly expressing MSCs exhibited increased expression of CCL2, a chemokine that recruits immune cells, while levels of the anti-inflammatory cytokine TGF-β were underexpressed. Notably, despite elevated IL-6 levels, immune cells stimulated under MLR and PHA conditions did not show an increase in Th17-derived IL-17A production. This suggests that MSC-derived IL-6 may modulate immune responses differently from IL-6 produced by immune cells, as it does not enhance Th17 activation or inflammation.

A key finding of our study was the significant suppression of RANKL expression in PHA-activated PBMCs by the ASC^IL-6H^, more pronounced than in the ASC^IL-6L^. This is particularly relevant to osteoarthritis, where RANKL promotes osteoclastogenesis and bone resorption [33]. RANKL has been shown to induce the expression of monokine induced by IFN-γ (MIG) in osteoclast precursors, facilitating adhesion and migration [34]. Additionally, IFN-γ increases RANKL in CD4+ T cells, contributing to bone loss in diseases like periodontitis [35]. 

Our findings are consistent with earlier studies showing that MSCs can suppress RANKL expression and inhibit osteoclastogenesis [36,37]. Importantly, ASC^IL-6H^ also more effectively suppresses IFN-γ and TNF-α, further reducing osteoclastogenic factors. This enhanced suppression of RANKL can be attributed to the anti-inflammatory properties of IL-6, which, when expressed by ASCs from OA patients, inhibit inflammatory cytokines and limit bone resorption. The differential effects of IL-6 high and low ASCs on immune regulation and osteoclastogenesis provide valuable insights into the potential for optimizing stem cell-based therapies. 

Our results suggest that IL-6 high ASCs may offer greater therapeutic benefits in osteoarthritis by not only promoting tissue regeneration but also by suppressing key factors like RANKL that drive bone resorption. Selecting ASCs with higher IL-6 expression could enhance the clinical efficacy of stem cell treatments for OA, particularly in reducing inflammation-mediated osteoclastogenesis. To further validate these findings, additional studies are necessary to determine whether this mechanism fully accounts for the observed reduction in bone degradation by osteoclasts. Additionally, further studies are needed to evaluate the direct immunomodulatory effects of ASCs based on IL-6 expression levels. Chondrocytes and ASCs could be co-cultured under experimental conditions simulating arthritis or exposure to pro-inflammatory molecules to investigate changes in the expression of chondroprotective and chondrolytic markers Future research should focus on evaluating the effects of IL-6 expression on cartilage regeneration in patients receiving clinical ASC treatments, as well as exploring how IL-6 expression in osteoclasts impacts the overall therapeutic efficacy of ASCs. These investigations will help clarify the role of IL-6 expression in shaping the therapeutic outcomes of ASC-based therapies.

## Figures and Tables

**Table 1:** Patients’ characteristics at the time of liposuction.

Donor No.	Age (Years)	Sex (F/M)	BMI	OA Symptom Duration (Months)	VAS Pain Score	K-L Grade
1	58	F	26.9	6	6	II
2	48	F	21.5	6	6	II
3	56	F	29.4	6	6	IV
4	60	F	22.7	5	4	II
5	59	M	25.1	7	5	IV
6	70	F	27.7	13	5	II
7	52	F	29.1	40	6	IV
8	62	F	33.0	18	7	III
9	63	M	28.1	10	5	II
10	67	F	26.4	16	5	III
11	55	M	26.9	180	5	II
12	67	F	23.0	2	7	III
13	65	F	24.9	6	5	III
14	57	M	27.0	27	6	II
15	60	M	27.8	6	5	III
16	64	F	26.9	2	5	IV
17	57	M	31.4	3	6	III
18	63	M	30.1	17	8	IV
19	58	M	26.6	26	6	III
20	59	M	30.6	3	6	III
21	63	F	27.2	27	4	II
22	62	F	26.4	2	7	II

## Data Availability

The data that support the findings of this study are available upon reasonable request.

## Supplementary Materials

The following supporting information can be downloaded at: https://www.mdpi.com/article/10.3390/cells13242046/s1, Figure S1. Differential IL-6Rα Expression in High and Low IL-6-Expressing ASCs
