# Supplementary material for: Elevated IL-6 Expression in Autologous Adipose-Derived Stem Cells Regulates RANKL Mediated Inflammation in Osteoarthritis
Source: Cells. 2024 Dec 11;13(24):2046. doi: 10.3390/cells13242046 (PMC11674629; doi:10.3390/cells13242046)
Supplement: Supplementary file 1 [file cells-13-02046-s001.zip › cells-3311540-supplementary.pdf]

IL-6Rα expression

> IL-6 high –ASCs

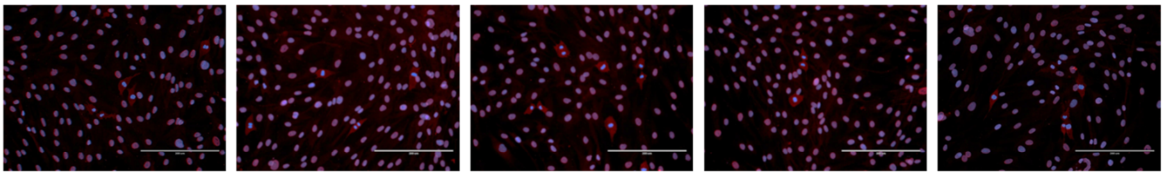

> IL-6 low –ASCs

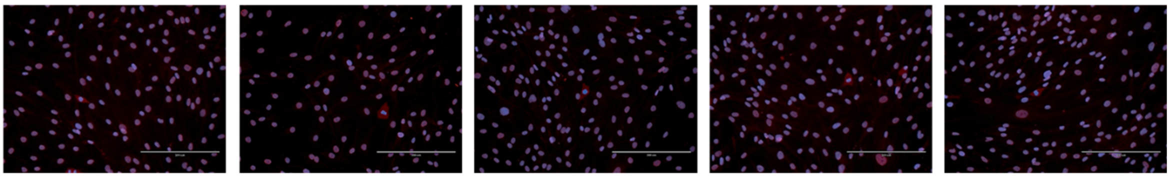

Supplementary figure S1. Differential IL-6Rα Expression in High and Low IL-6-Expressing ASCs
